# Bacterial Distribution in the Glacier Borehole Meltwater on the Eastern Broknes Peninsula of the Larsemann Hills and Adjacent Lake Water, East Antarctica

**DOI:** 10.3390/microorganisms13030679

**Published:** 2025-03-18

**Authors:** Hongpeng Cui, Jibin Han, Bing Li, Youhong Sun, Da Gong, Xiaopeng Fan, Talalay Pavel, Dayi Zhang, Liang Gao, Hongchen Jiang

**Affiliations:** 1 School of Ocean Sciences, China University of Geosciences (Beijing), Beijing 100083, China; cuihongpeng123@163.com (H.C.); lgao@live.cn (L.G.); 2 Key Laboratory of Polar Geology and Marine Mineral Resources, Ministry of Education, China University of Geosciences (Beijing), Beijing 100083, China; 3 Hainan Institute, China University of Geosciences (Beijing), Sanya 572000, China; bing@jlu.edu.cn (B.L.); syh@cugb.edu.cn (Y.S.); 4 Qinghai Institute of Salt Lakes, Chinese Academy of Sciences, Xining 810008, China; jbhan@isl.ac.cn; 5 School of Engineering and Technology, China University of Geosciences (Beijing), Beijing 100083, China; 6 Polar Research Centre, Jilin University, Changchun 130061, China; gongda@jlu.edu.cn (D.G.); heaxe@163.com (X.F.); ptalalay@yandex.ru (T.P.); 7 Key Laboratory of Groundwater Resources and Environment Ministry of Education, Jilin University, Changchun 130021, China; zhangdayi@jlu.edu.cn

**Keywords:** bacterial community, lakes, glacier, community assembly, Larsemann Hills, Antarctic

## Abstract

The distribution and assembly mechanisms of microorganisms in Antarctic lakes and glaciers remain poorly understood, despite their ecological significance. This study investigates the bacterial diversity and community composition in glacier borehole meltwater samples from the eastern Broknes Peninsula of the Larsemann Hills and adjacent lake water samples in East Antarctica using high—throughput 16S rRNA gene sequencing. The results show that bacterial diversity in glacier borehole meltwater increased with depth, but remained lower than in lake water. Significant compositional differences were observed between lake and glacier borehole bacterial communities, with higher relative abundances of *Actinobacteria*, *Bacteroidia*, *Cyanobacteriia,* and *Verrucomicrobiae* in glacier borehole water samples, while *Alphaproteobacteria*, *Gammaproteobacteria*, *OLB14* (phylum *Chloroflexi*), *Acidimicrobiia*, and *Thermoleophilia* were more abundant in lake samples. These differences were attributed to distinct community assembly mechanisms: stochastic processes (ecological drift and dispersal limitation) dominated in lakes, while both stochastic (ecological drift and homogeneous dispersal) and deterministic (homogeneous selection) processes played key roles in glacier boreholes. This study enhances our understanding of bacterial community assembly and distribution patterns in Antarctic glacier ecosystems, providing insights into microbial biodiversity and biogeochemical cycling in these extreme environments.

## 1. Introduction

Antarctica represents one of the harshest environments on the planet, characterized by formidable winds, frigid temperatures, elevated radiation levels, and limited water availability. These extreme environmental stressors impose significant constraints on biological survival and proliferation of life [1,2,3,4,5]. Microbial consortia function as keystone taxa in Antarctic ecosystems, playing a crucial role in the carbon cycle as well as in the movement of nutrients and energy [6,7]. Glaciers represent a quintessential ecosystem in polar regions, dominated by bacteria and algae that drive biogeochemical processes [8]. Microorganisms originating from atmospheric deposition (encompassing both wet and dry deposition) settle upon the surfaces of glaciers and initiate colonization as the glaciers undergo compaction processes [9,10,11]. The composition of these microbial communities is intricately linked to their ecological functions, thereby regulating nutrient cycling and energy dynamics [12,13,14]. Nonetheless, the distribution patterns of microorganisms and the assembly mechanisms of the microbial communities that inhabit glaciers during the compaction process remain inadequately characterized. Further investigation is imperative to elucidate these processes and their broader implications for the functionality of Antarctic ecosystems.

Polar glaciers harbor diverse lacustrine systems exhibiting significant variations in geomorphometric parameters (e.g., age, size, depth), thermal regimes, biogeochemical processes, and ionic compositions. These lakes may be blanketed by ice on a seasonal basis or persistently throughout the year, and are often situated upon ice—free surfaces [15,16]. Extensive research has been devoted to exploring various dimensions of these glacial lakes, encompassing microbial diversity, biogeochemical processes, ultraviolet resistance mechanisms, survival strategies, and microbial responses to climate change and anthropogenic influences [7,17,18,19,20,21,22,23,24,25,26,27]. These lakes, serving as pivotal sites of atmospheric microbial deposition, represent a critical zone for microbial succession upon glaciers. Moreover, nitrogen and methane cycles have been recognized as integral processes within the climate system of Antarctic glaciers [28]. Recent investigations have underscored the importance of zinc (Zn) as an essential nutrient supporting primary production in the Southern Ocean, wielding considerable impact on the biogeochemical interactions of the Antarctic atmosphere—land—ocean continuum [29,30]. Nonetheless, there remains a significant gap in our understanding of the microorganisms implicated in the geochemical cycling of elements within Antarctic lakes and glaciers, highlighting the necessity for further inquiry.

The assembly of microbial communities in Antarctic ecosystems is governed by a complex interplay of deterministic and stochastic processes. Deterministic processes include mechanisms such as variable and homogeneous selection, whereas stochastic processes encompass phenomena like homogenization diffusion, diffusion limitation, and ecological drift [31,32,33]. Recent studies have shed light on the factors influencing microbial community assembly across different Antarctic habitats, highlighting significant variations in the key controlling factors. For example, research indicates that the formation of bacterial communities at the leading edge of Antarctic glaciers is primarily influenced by deterministic processes [34]. In contrast, studies of surface sediments in the Ross Sea reveal a predominance of stochastic processes in shaping microbial communities [35]. Additionally, in the degraded glacial soils of the Antarctic Ocean, bacterial community dynamics are largely controlled by deterministic factors, while fungal community structures tend to be more influenced by stochastic processes [36]. Despite these insights, the mechanisms driving the assembly of microbial communities as they transition from surface lake environments to deeper glacial settings remain inadequately understood. This knowledge gap underscores the need for further research to elucidate the underlying processes and their implications for microbial diversity, ecosystem functioning, and biogeochemical cycles in Antarctic ecosystems.

To address the above issues, we utilized data obtained from the 38th Antarctic Scientific Expedition, during which we collected meltwater samples from glacial boreholes at three distinct depths within the eastern Larsemann Hills of southeast Antarctica. Additionally, we gathered surface water samples from four nearby lakes. The bacterial community composition in these samples was analyzed using high—throughput sequencing technologies. This approach allowed us to gain valuable insights into microbial succession patterns and assembly mechanisms that regulate microbial communities in Antarctic glaciers. By comparing microbial communities at varying depths in glacial meltwater and contrasting these with those from surface lakes, we aimed to elucidate the influences of environmental factors and ecological processes on community structure and dynamics in this unique and extreme environment. These findings are expected to contribute to a better understanding of polar microbial biodiversity, biogeochemical cycling, and the potential impacts of climate change in Antarctic ecosystems.

## 2. Materials and Methods

### 2.1. Study Site and Sample Collection

The Larsemann Hills, located on the east coast of Antarctica, are recognized as the second largest ice—free oasis in the region [37]. This unique area contains two significant peninsulas: the Broknes Peninsula to the east and the Stones Peninsula to the west. Within this oasis, there are approximately 150 freshwater lakes [38,39], which present a diverse range of sizes and ecological characteristics. These lakes can be categorized as either seasonal or perennial. Specifically, some of these lakes experience brief periods without ice cover, while others remain ice—free during the summer months from December to February. During the summer, temperatures in these lakes can rise to around 8 °C [40,41]. 

**Figure 1:**
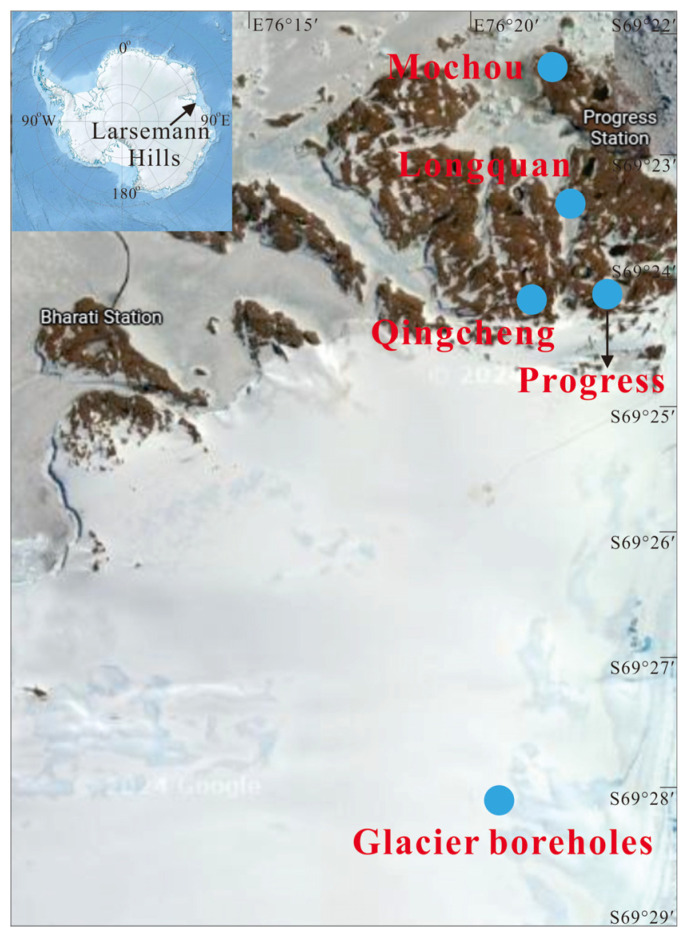
Map showing the study area and sample locations.

During January 2022, the Chinese National Antarctic Research Expedition designated the Broknes Peninsula within the East Antarctic Rasman Mountains as the focus of their scientific inquiry. The study area encompassed four meticulously selected lakes—Mochou Lake, Longquan Lake, Jinbu Lake, and Qingcheng Lake—alongside adjacent ice boreholes (Figure 1). The mean temperature during the sampling period ranged from 4 °C to 10 °C, creating an intriguing environment for the study. Water samples were systematically collected from each of the four lakes, while three boreholes were drilled south of Progress Lake utilizing a hot melt drill to reach depths of 135.3 m, 183 m, and 200 m beneath the ice surface, which. Pollution—free thermal melting detectors facilitated the collection of subglacial meltwater [42]. Aseptic techniques were employed to ensure the collection of approximately 500 mL of water samples and meltwater from each lake, as well as from the three selected depths of the boreholes. The precise coordinates of the sampling sites are detailed in Table S1. To ensure the integrity of the samples, both lake and meltwater were preserved in sterile polypropylene bottles and maintained at −20 °C both on site and during transit. Upon arrival at the Geomicrobiology Laboratory of the China University of Geosciences in Beijing, the samples were further stored at −20 °C until they could undergo analysis.

### 2.2. Water Chemistry Measurements

Concentrations of major nutrients (F^−^, Cl^−^, NO_2_^−^, SO_4_^2−^, Br^−^, NO_3_^−^, Na^+^, K^+^, Mg^2+^ and Ca^2+^) were analyzed in lake and glacier borehole meltwater samples using ion chromatography ICS—600 (Thermo Fisher Scientific, Waltham, MA, USA). The δD and δ^18^O values of the samples were determined using a liquid water isotope analyzer (LGR Corporation, Mountain View, CA, USA). Forty—three dissolved trace elements (e.g., Li, Be, As, Cu, Pb, Zn, etc.) were analyzed in water and glacier borehole meltwater samples using inductively coupled plasma mass spectrometry (ICP—MS, PE 350D) at the State Key Laboratory of Biogeology and Environmental Geology of the China University of Geosciences, Wuhan, China. [43,44].

### 2.3. DNA Extraction and Polymerase Chain Reaction (PCR) Amplification

Fifty milliliters of water sample in three replicates was filtered through a 0.22 μm membrane. The resulting filter membrane containing the biomass was then used for DNA extraction. DNA extraction was performed using the FastDNA SPIN Kit for Soil (MP Biomedicals, Santa Ana, CA, USA). The quality and quantity of DNA were assessed using the Nanodrop fluorescence method (Thermo Scientific, NanoDrop ND—1000). PCR amplification of the bacterial 16S rRNA gene was performed by polymerase chain reaction (PCR) based on the MiSeq Illumina platform (Illumina, San Diego, CA, USA) using the bacterial primer set of 338F (5′—barcode—ACTCCTACGGGAGGCAGCAG—3′)/806R (5′—GGACTACHVGGGTWTCTAAT—3′) [45]. Barcodes were used to differentiate the samples. PCR reactions were performed in three replicates using a composite DNA template, and the PCR products from the three replicates were mixed and examined by gel electrophoresis. Each PCR reaction was performed in a 20 μL reaction mixture containing 2.5 μL of 10× Ex Taq buffer, 2 μL of 2.5 mM dNTPs, 0.5 μL of each primer, 0.25 μL of Ex Taq polymerase (Ex Taq; TaKaRa, Dalian, China), and approximately 10 ng of DNA. The PCR amplification program was: initial denaturation for 3 min at 95 °C; 27 cycles of 30 s at 95 °C; 30 s at 55 °C; 45 s at 72 °C; and a final extension of 10 min at 72 °C [46].

### 2.4. Sequencing and Bioinformatics

For taxonomic classification, sequences were clustered into operational taxonomic units (OTUs) at a 3% level of dissimilarity, and a representative sequence for each OTU was selected using the RDP classifier at a threshold of 70% and assigned to the SILVA 16S rRNA database [47]. Coverage for all samples was >0.99. Alpha diversity parameters (Ace, Chao1, Shannon and Invsimpson) were calculated in MOTHUR (version 1.30.2) [48]. Principal Coordinate Analysis (PCoA) was performed to measure community similarity using Bray–Curtis distance. An analysis of one—way similarity (ANOSIM) was conducted in PRIMER 5 to test whether the spatial separation of different samples in the PCoA scoring plots was statistically significant. Welch’s *t*—test was used to reveal which organisms were the main contributors to differences in community composition between clusters. Spearman’s rank correlations were calculated to analyze the relationship between bacterial clusters and environmental parameters. In addition, Spearman’s rank coefficients (r) between relative abundances of genera were calculated using the “picante” package with a significance level of *p* < 0.05 and a correlation coefficient of |r|> 0.6 in the R environment (version 3.6.2). Sequence data generated in this study have been deposited in the NCBI Sequence Read Archive (SRA) database under the accession number: PRJNA1090395.

To compare community assembly processes between lake and glacier borehole meltwater samples, paired β nearest taxon indices (βNTI) were calculated using the R package picante [49]. βNTI calculations quantify the number of standard deviations between the observed mean β nearest taxon distance (βMNTD) and the mean of the null distribution. If |βNTI| > 2, this indicates that species phylogenies are clustered or phylogenies are overdispersed, and that differences in microbial community composition are primarily caused by deterministic processes, if |βNTI| < 2, the effect of stochastic assembly on microbial community composition is more pronounced. Further comparisons were made by βNTI and the Bray–Curtis based Raup–Crick metric (RCbray) to assess assembly mechanisms, including homogeneous selection (βNTI < −2), heterogeneous selection (βNTI > 2), dispersal limitation (|βNTI| < 2 and RCbray > 0.95), homogenizing dispersal (|βNTI|< 2 and RCbray < −0.95), and ecological drift (undominated fraction) (|βNTI| < 2 and |RCbray| < 0.95).

## 3. Results

### 3.1. Environmental Parameters

The concentrations of nutrients in the seven samples are presented in Table S2. Among the lake water samples, Mochou Lake exhibits the highest levels of major nutrients (excluding NO_3_^−^). Cl^−^, NO_2_^−^, SO_4_^2−^, Na^+^, and Mg^2+^ were detected across all four lakes. In contrast, for the glacier borehole meltwater samples, SO_4_^2−^ and NO_3_^−^ were observed in three samples at depths of 135.5 mbsf, 183 mbsf, and 200 mbsf, ranging from 0.17 ppm to 1.13 ppm and 0.07 ppm to 1.11ppm, respectively. In general, the glacier borehole meltwater samples are characterized by lower δD and δ^18^O values than those of the lake water samples, with δD and δ^18^O values decreasing with depth within the glacier borehole, ranging from −319.40‰ to −162.58‰ and −40.02‰ to −21.20‰, respectively. Furthermore, Table S3 provides the concentrations of 43 dissolved trace elements, including but not limited to Li, Be, As, Cu, Pb, and Zn, in both the lake and glacier borehole meltwater samples.

### 3.2. Alpha (α) Diversity Indices

A total of 309,947 bacterial sequences were successfully obtained from seven different samples, as summarized in Table 1. For the lake samples, the number of bacterial operational taxonomic units (OTUs) ranged from 174 to 256 with an average of 218. The alpha diversity indices calculated for the lake samples, including the Ace, Chao 1, Shannon, and Invsimpson indices, varied as follows: Ace (190.18 to 285.36, 237.44 on average), Chao 1 (189.12 to 281.24, 235.36 on average), Shannon (2.65 to 2.82, 2.78 on average), and Invsimpson (6.96 to 11.59, 9.55 on average). In comparison, the glacier borehole meltwater samples revealed a different pattern in terms of bacterial diversity. The number of bacterial OTUs ranged from 92 to 141, with an average of 116, which is notably lower than that observed in the lake samples. The alpha diversity indices for the glacier borehole meltwater samples showed the following ranges: Ace (102.69 to 145.14, 123.35 on average), Chao 1 (103.00 to 147.43, 124.29 on average), Shannon (2.89 to 3.00, 2.93 on average), and Invsimpson (10.08 to 11.20, 10.59 on average). Importantly, a trend was observed in the glacier borehole meltwater samples, where the number of OTUs, as well as the Ace and Chao 1 indices, increased with the depth of the boreholes. This suggests that deeper layers may harbor more bacterial community richness, potentially influenced by variations in environmental conditions, nutrient availability, or ecological interactions as depth increases.

### 3.3. Bacterial Diversity

**Figure 2:**
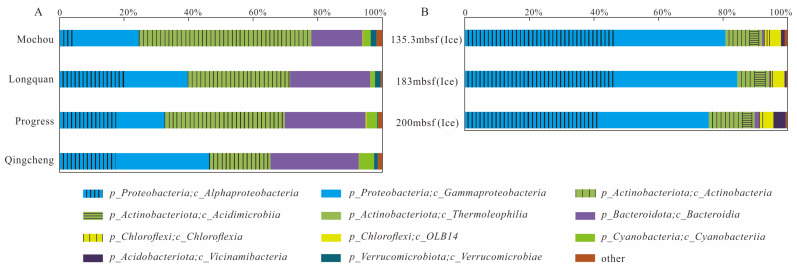
Relative abundance of major bacterial groups (>1%, at the class level) in the seven samples from the lakes and glacier borehole meltwater. (**A**) Total of bacterial groups; (**B**) composition of bacterial groups.

Figure 2 summarizes the composition of bacterial communities at the order level for the seven samples. In the lake samples, the main bacterial orders consisted of *Burkholderiales* (14.65–28.93%, dominated by the class *Gammaproteobacteria*), *Frankiales* (18.88–49.97%, dominated by the class *Actinobacteria*), *Sphingomonadales* (1.14–6.01%) and *Acetobacterales* (0.08–15.48%) belonging to the class *Alphaproteobacteria*, and *Cytophagales* (2.28–13.43%), *Chitinophagales* (0.45–12.24%) and *Sphingobacteriales* (1.31–11.01%), which belong to the class *Bacteroidia*. In the glacier borehole meltwater samples, the dominant bacterial orders were *Sphingomonadales* (29.93–37.10%) and *Rhizobiales* (5.82–10.51%) belonging to the class of *Alphaproteobacteria*, and *Burkholderiales* (21.17–32.18%) and *Pseudomonadales* (1.60–13.91%) belonging to the class of *Gammaproteobacteria*. Furthermore, *Propionibacteriales* (3.70–8.26%), *norank_OLB14* (3.24–3.66%), and *Microtrichales* (2.75–3.16%) dominated the bacterial community, belonging to the class of *Actinobacteria*, *OLB14* (phylum *Chloroflexi*) and *Acidimicrobiia*, respectively.

**Figure 3:**
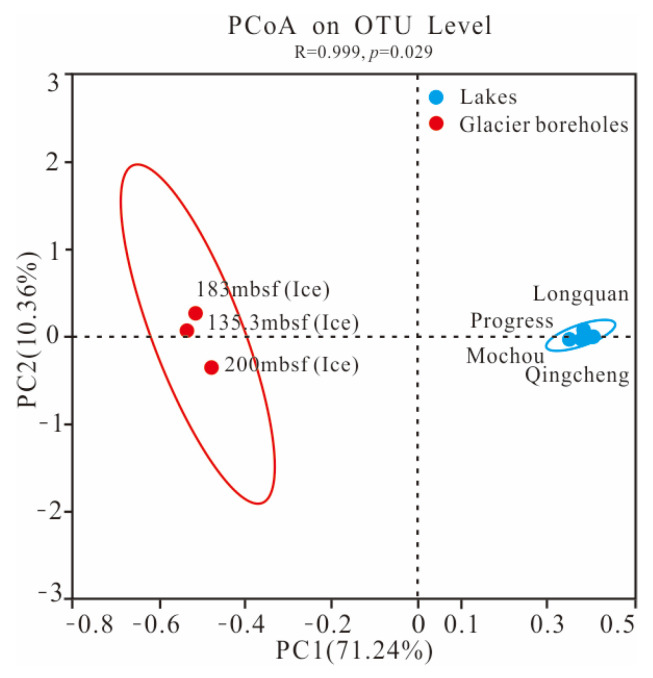
The PCoA plot showing the difference in bacterial community structure among the seven samples from the lakes and glacier borehole meltwater using the Bray—Curtis distance based on the OTU level. Ellipses represent 95% confidence intervals.

**Figure 4:**
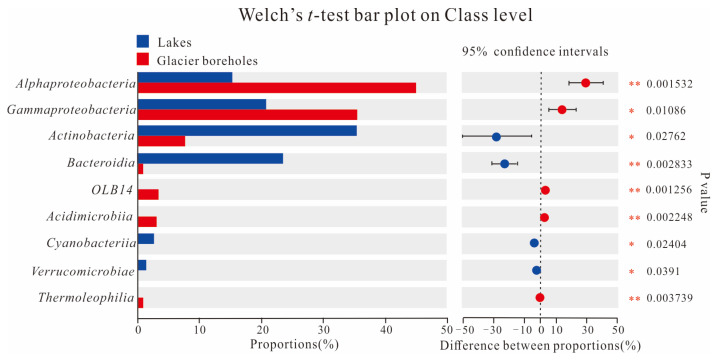
Welch’s *t*—test showing significant differences in the bacterial communities at class level among the samples from the lake and glacier borehole meltwater. Statistical significance indicates by asterisks: * 0.01 < *p* value ≤ 0.05; ** 0.001 < *p* value ≤ 0.01).

The PCoA score plot revealed that the bacterial community structure in lake water was significantly different from that in glacier borehole meltwater (Figure 3). The relative abundances of *Actinobacteria* (35.36%), *Bacteroidia* (23.31%), *Cyanobacteriia* (2.95%), and *Verrucomicrobiae* (1.26%) were significantly higher in the glacier borehole meltwater than in the lake water samples (Figure 4), while the relative abundances of *Alphaproteobacteria* (44.93%), *Gammaproteobacteria* (35.43%), *OLB14* (3.47%, phylum *Chloroflexi*), *Acidimicrobiia* (3.13%), and *Thermoleophilia* (1.00%) showed an opposite trend (Welch’s *t*—test, *p* < 0.05). Moreover, Welch’s *t*—test analysis also indicated that significant differences in the bacterial community at the genus level were found between the lake water samples and glacier borehole meltwater samples (*p* < 0.05) (Figure S1). The abundance of genera *Massilia* and *Actimicrobium* (20.42% and 3.35%, order *Burkholderiales*), *norank_OLB14* (3.47%, phylum *Chloroflexi*), *Methylobacterium*—*Methylorubrum* (2.82%, order *Rhizobiales*), *Iamia* and *Ilumatobacter* (1.46% and 1.24%, order *Microtrichales*), as well as *norank_67*—*14* (0.94%, order *Solirubrobacterales*) was significantly higher in the glacier borehole meltwater samples than that in lake samples, while the relative abundances of *unclassified_Sporichthyaceae* and *hgcI_clade* (20.05% and 14.06%, order *Frankiales,* known as the typical freshwater bacterial group), *Polynucleobacter* and *Polaromonas* (8.93% and 6.49%, order *Burkholderiales*) showed an opposite trend (Welch’s *t*—test, *p* < 0.05) (Figure S1).

### 3.4. Correlation Analysis of Bacterial Communities with Environmental Factors

**Figure 5:**
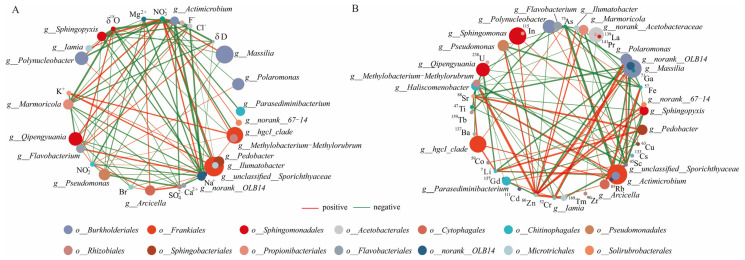
Spearman’s correlation analysis between the genera bacterial groups and environmental factors of the lake and borehole samples. A connection stands for a strong (Spearman’s |r|> 0.6) and significant (*p* < 0.05) correlation. Red and green edges indicate positive and negative correlations, respectively. (**A**) Major nutrients. (**B**) Dissolved trace elements.

In general, the major nutrients NO_2_^−^, NO_3_^−^, Na^+^, K^+^, and the dissolved trace elements Li, As, Rb, Sr, and Sc correlated significantly with the characterized bacterial genera in the lake samples (*unclassified__Sporichthyaceae*, *hgcI_clade*, *Polaromonas,* and *Polynucleobacter*) (Figure 5). NO_2_^−^ exhibited a positive correlation with these characterized genera (except *Polynucleobacter*) and the dominant bacterial genera *Arcicella* and *Flavobacterium*. In the glacier borehole meltwater samples, NO_3_^−^ was the only major nutrient that showed a positive correlation with the characterized bacterial genera (*Massilia*, *Actimicrobium*, *norank_OLB14*, *Methylobacterium*—*Methylorubrum*, *Iamia*, *Ilumatobacter,* and *norank_67*—*14*), as well as with the dominant bacterial genera *Qipengyuania*, *Marmoricola*, *Sphingopyxis,* and *Bosea*. Zn was the only dissolved trace element that showed a positive correlation with these genera (except *Actimicrobium*) and the dominant bacterial genera (*Sphingomonas*, *Pseudomonas*, *Marmoricola*, *Sphingopyxis,* and *Bosea*). Moreover, most characterized bacterial genera (except for *norank_67–14*) exhibit negative correlations with the δ^18^O value, while *Actimicrobium* and *norank_OLB14* (phylum *Chloroflexi*) displayed negative correlations with the δD value in the glacier borehole meltwater.

### 3.5. Stochastic and Deterministic Assembly Processes

**Figure 6:**
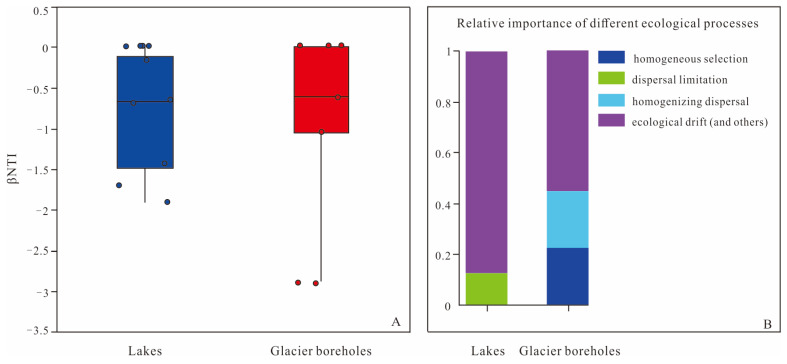
Assembly of the bacterial communities of the lake and glacier borehole meltwater. (**A**) Distribution of β nearest taxon index (βNTI) among different samples. Blue-colored and Red-colored symbols indicate the lake and glacier borehole meltwater samples, respectively. (**B**) Contributions of ecological processes to microbial bacterial assembly.

The analysis of ecological processes in this study revealed that the interplay between deterministic and stochastic processes contributed to the structural variations observed in bacterial communities from glacier borehole meltwater and lake water samples (Figure 6). Most βNTI values across all samples were found to be between −2 and +2, which indicates a significant influence of stochasticity in the assembly of these microbial communities. Specifically, for bacterial community assembly in lake water, stochastic processes were predominantly driven by ecological drift (87.5%) and dispersal limitation (12.5%). In contrast, the bacterial community assembly in glacier borehole meltwater was similarly influenced by stochastic processes, but the contributions differed slightly: ecological drift accounted for 55.56% of the assembly processes, while homogenizing dispersal contributed 22.22%.

## 4. Discussion

### 4.1. Comparative Analyses of Bacterial Communities Between the Lakes and Glacier Boreholes

Generally, the Antarctic lakes contain a majority of *Proteobacteria*, *Actinobacteria*, *Cyanobacteria,* and *Bacteroidetes,* and less abundant phyla include *Chloroflexi*, *Acidobacteria*, and *Firmicutes*. Moreover, *Firmicutes* might be more dominant in the glacier ice [22,25,28]. In this study, the phylum of *Proteobacteria*, *Actinobacteriota*, *Bacteroidota*, and *Chloroflexi* occupied an absolute proportion of the bacterial groups (~90%). Marked disparities in bacterial communities were observed between lake and glacier environments. The relative abundances of *unclassified Sporichthyaceae* and *hgcI_clade* (order *Frankiales*), as well as *Polynucleobacter* and *Polaromonas* (order *Burkholderiales*), were significantly greater in the lake samples compared to those from glacier borehole meltwater at the genus level (*p* < 0.05) (Figure S1). *Frankiales* are often associated with actinorhizal plants and possess the capability to fix nitrogen, which has been previously identified in Arctic ecosystems [50,51]. The genera *Polynucleobacter* and *Polaromonas,* prevalent in the lake samples, are reported to be ubiquitous in the oligotrophic lakes of Antarctica [28]. Members of the order *Burkholderiales* have also been identified in Arctic sediments characterized by low organic carbon content [52]. These groups encompass taxa known for their ability to degrade petroleum hydrocarbons, suggesting that they play a vital role in initial colonization processes through nitrogen fixation [53,54]. In this study, the four characterized bacterial genera—including *unclassified Sporichthyaceae*, *hgcI_clade* (order *Frankiales*), *Polynucleobacter*, and *Polaromonas* (order *Burkholderiales*) that were significantly enriched in the lake samples—exhibited negative correlations with nitrate (NO_3_^−^) (*p* < 0.05). In contrast, most of these genera, with the exception of *Polynucleobacter*, showed positive correlations with nitrite (NO_2_⁻) (*p* < 0.05) (Figure 5). These findings suggest that these four bacterial groups may play a significant role in the nitrogen cycling processes within the lakes. Specifically, it is likely that *Polaromonas*, along with *unclassified Sporichthyaceae* and *hgcI_clade*, is actively involved in denitrification processes. The *hgcI_clade*, the typical freshwater bacterial group, is capable of both heterotrophic and autotrophic lifestyles, and can dissolve organic carbon in low—temperature water [55,56]. This indicates the complexity of the microbial nitrogen cycle in Antarctic cryogenic lake water, and a possible functional interplay in nitrogen transformations, where these bacteria could contribute to the conversion of nitrates into nitrogen gas or other nitrogenous compounds, thereby influencing the nitrogen dynamics in the lake ecosystems.

The abundance of the genera *Massilia* and *Actimicrobium* (order *Burkholderiales*), *norank_OLB14* (phylum *Chloroflexi*), *Methylobacterium*—*Methylorubrum* (order *Rhizobiales*), *Iamia*, and *Ilumatobacter* (order *Microtrichales*), as well as *norank_67—14* (order *Solirubrobacterales*), was significantly higher in glacier borehole meltwater samples compared to lake samples (*p* < 0.05) (Figure S1). The characteristics of these seven genera suggest that they may have adapted to cold environments. *Actimicrobium*, which has rarely been documented in Antarctic glaciers, includes *Actimicrobium antarcticum*, a psychrophilic and facultatively anaerobic bacterium isolated from Antarctic seawater [57]. Members of *Microtrichales* have been shown to play an essential role in the heterotrophic mineralization of carbon within polar and temperate sediment ecosystems [58]. On the other hand, organisms from *Solirubrobacterales* are known for their ability to degrade plant polymers, such as lignin and cellulose, through various extracellular hydrolytic enzymes, which allows them to persist in cold, nutrient—poor environments like Antarctica’s Dry Valleys [59,60]. *Rhizobiales* are recognized for their nitrogen—fixing capabilities as plant symbionts, and studies indicate that their relative abundance typically increases in eutrophic waters [61,62]. Furthermore, Spearman’s correlation analysis revealed that each of these seven characterized genera showed positive correlations with nitrate (NO_3_⁻) (*p* < 0.05) (Figure 5), indicating a potential link to nitrification processes in Antarctic glaciers. In alignment with this, *Burkholderiales* have been noted for their active involvement in the oxidation of ammonium (NH₄⁺) to nitrate (NO_3_⁻) [53,63]. Additionally, *OLB14* refers to a bacterial strain of the taxonomic group *Chloroflexi*, usually presenting as *Cloroflexi bacterium OLB14*, and from that moment on the abbreviation was used. These groups have often been observed in oligotrophic environments, such as partial nitrification—anammox systems, and may participate in nitrate respiration through partial denitrification [64,65].

### 4.2. Bacterial Distribution Changed with Glacier Borehole Depth

The variation of hydrogen and oxygen isotope ratios in the waters of Antarctic lakes and glacier borehole meltwater provides valuable insights into climate and environmental changes influencing glacier development. In this study, the ratios of δD and δ^18^O in the lakes were found to be high, indicating an enrichment of lighter isotopes. In contrast, the meltwater in glacier boreholes exhibited depleted heavy hydrogen and oxygen isotope values (Table S2). The behavior of light and heavy isotopes is influenced by temperature, with lighter isotopes favoring condensation into ice in colder environments, while heavier isotopes are more likely to condense into water vapor or liquid water during warmer conditions [66,67,68]. Consequently, the study observed that δD and δ^18^O depletions increased with depth from the surface water of the lakes to the glacier, suggesting a trend of increasing paleoatmospheric temperature since the formation of the glacier above the studied borehole depths. However, it is important to acknowledge that the low sample accuracy warrants further confirmation of this temperature change trend. Interestingly, different microbial groups respond differently to rising temperatures. As the temperature increased during the formation and thickening of the ice layer, certain characterized bacterial genera, including *Massilia* and *Actimicrobium*, as well as other groups (e.g., *Qipengyuania*, *Marmoricola*, and *Sphingopyxis*), showed a decrease in relative abundance from depths of 200 m below the surface (mbsf) to 135.3 mbsf. Conversely, other characterized genera, including *norank_OLB14* (phylum *Chloroflexi*), *Methylobacterium–Methylorubrum*, *Iamia*, *Ilumatobacter*, and *norank_67*—*14*, along with groups like *Sphingomonas* and *Pseudomonas*, exhibited an increasing trend in relative abundance within the same depth range (Figure S2). This differential response highlights the complexity of microbial communities in relation to environmental changes and temperature fluctuations in these extreme ecosystems.

Furthermore, all characterized genera, with the exception of *Actimicrobium*, exhibited significantly high abundance in the glacier borehole meltwater samples, demonstrating positive correlations with element Zn (*p* < 0.05). This correlation was notably absent in the lake samples. Conversely, two characterized bacterial genera, *Polynucleobacter* and *Polaromonas*, displayed negative correlations Zn^2+^. Glaciers serve as natural archives that retain temporally structured information about climate and ecosystems, including microbial communities [69]. Zinc, recognized as a critical trace nutrient in marine environments, plays a pivotal role in influencing primary productivity, particularly in polar regions [29]. Therefore, the observed positive correlation between microorganisms and Zn^2+^ in the glacier boreholes implies that the zinc cycle may exhibit heightened sensitivity to the assembly mechanisms of the microbial community, as well as to the biogeochemical cycling of metal elements, particularly in the context of climate—driven changes in Antarctic glacial environments.

### 4.3. Assembly Processes for the Samples from the Lakes and Glacier Boreholes

While acknowledging the limitation of single—day sampling (January 2022) in capturing seasonal or annual microbial dynamics, our study maintains methodological validity due to three key factors: (1) strategic timing during peak melt season ensures more microbial activity and chemical stability observed in preliminary December–February field data; (2) physicochemical parameters (e.g., δD/δ^18^O) remained stable within the sampling week (<0.5‰ variation), largely reducing transient effects; and (3) the extreme aridity of Larsemann Hills (<200 mm annual precipitation) reduces stochastic environmental perturbation. These factors collectively support the representativeness of our findings for Antarctic glacial lake systems. Future studies should integrate multi—seasonal surveys and high—frequency monitoring to address temporal variability and advance mechanistic understanding of microbial assembly processes in polar environments.

To quantify the relative contributions of determinism versus stochasticity in the assembly of bacterial communities, βNTI (beta nearest taxon index) values were calculated for both lake and glacier borehole meltwater samples. Deterministic processes in community assembly primarily involve selection mechanisms influenced by species characteristics, species interactions, and environmental conditions. In contrast, stochastic processes are driven by random events related to birth, death, colonization, extinction, and speciation [32]. In various Antarctic environments, deterministic processes are generally recognized as the predominant drivers of bacterial community assembly. For example, studies have highlighted their significance in locations such as the glacier front of Anvers Island, the lakes in the Vestfold Hills, and recently deglaciated seabeds [34,36,70]. These deterministic processes may include selective pressures resulting from temperature gradients, nutrient availability, and physical habitat characteristics, which collectively shape the community structure. On the other hand, stochastic processes have been identified as the primary driving forces for community assembly in different contexts, such as the surface sediments of the Ross Sea, where random environmental dynamics might play a more critical role [35]. This suggests that while deterministic factors are crucial in certain habitats, stochasticity can also significantly influence bacterial community dynamics in more variable or disturbed environments.

In this study, the βNTI values calculated for lake water samples ranged from −2 to 2, indicating that bacterial community assembly was primarily dominated by stochastic processes, particularly ecological drift (87.5%) and dispersal limitation (12.5%). These findings suggest that ecological drift and dispersal constraints significantly shaped the development of bacterial communities in these lake environments. In contrast, the bacterial community assembly in glacier borehole meltwater exhibited a more complex influence from both stochastic and deterministic processes. Specifically, ecological drift (55.56%) and homogenizing dispersal (22.22%) were significant stochastic processes, while deterministic processes, mainly homogeneous selection, accounted for the remaining 22.22%. The high proportion of ecological drift in both lake and glacier borehole samples may indicate the influence of additional processes, as outlined in the framework by Stegen et al. [49], including local adaptation to environmental heterogeneity or historical contingency. Dispersal limitation has a pronounced impact on abundant taxa, particularly those with large niche breadths, as suggested by Pandit et al. [71] and Wu et al. [72]. Taxa capable of reaching multiple locations are susceptible to these limitations, which can explain minor variations in the composition and structure of bacterial communities in the lakes. Dispersal limitation has also been highlighted as a key factor in community assembly in different environments, such as the sediments of the Ross Sea [35]. Homogenizing dispersal contributes to compositional similarity within communities, driven by high rates of dispersal, which may also significantly influence community structure [73,74]. In the glacier boreholes, this homogenizing dispersal, paired with homogeneous selection, may lead to community convergence. This convergence is likely due to a consistent selective environment, such as the extensive glacial layer present in these boreholes. Consequently, both homogenizing dispersal and homogeneous selection appear to play crucial roles in shaping community turnover and coexistence patterns in the glacial meltwater environments, pointing to complex interactions between stochastic and deterministic processes in bacterial community assembly.

## 5. Conclusions

Our research revealed significant differences in the composition of bacterial communities between glacier borehole meltwater and the adjacent lake water in the Larsemann Hills of Antarctica. Notably, the relative abundance of specific bacterial taxa within the glacier boreholes exhibited distinct increases and decreases correlating with variations in hydrogen and oxygen isotope values as depth increased. These changes in isotope ratios are indicative of fluctuations in paleoclimate conditions that occurred during the ice formation process. The observed distribution of bacterial communities in these contrasting environments is governed by different assembly mechanisms. In the lakes, stochastic processes are predominant in shaping bacterial community development. Conversely, within the glacier boreholes, community assembly appears to be influenced by a combination of both stochastic and deterministic processes. Our study contributes valuable insights into the evolutionary patterns and ecological assembly processes of microbial communities in the lakes of the Larsemann Hills and the nearby glaciers of eastern Antarctica. By highlighting how environmental factors and community assembly mechanisms interact, this research enhances our understanding of microbial dynamics in polar environments, offering potential implications for predicting how these communities might respond to ongoing climate changes.

## Figures and Tables

**Table 1:** Diversity indices of bacterial 16S rRNA genes in the lake water and glacier borehole meltwater samples.

Sites\Estimators	OTU	Ace	Chao 1	Shannon	Invsimpson
Mochou Lake	244	285.36	281.24	2.65	6.96
Longquan Lake	174	190.18	189.12	2.83	10.29
Progress Lake	199	202.77	204.50	2.82	9.36
Qingcheng Lake	256	271.44	266.56	2.82	11.59
135.3 mbsf (Ice)	92	102.69	103.00	2.89	10.48
183 mbsf (Ice)	116	122.21	122.43	3.00	11.20
200 mbsf (Ice)	141	145.14	147.43	2.89	10.08

Note: mbsf: abbreviation for meters below the surface.

## Data Availability

The original contributions presented in this study are included in the article/Supplementary Materials. Further inquiries can be directed to the corresponding author.

## Supplementary Materials

The following supporting information can be downloaded at: https://www.mdpi.com/article/10.3390/microorganisms13030679/s1, Table S1: Locations of sampling sites of the lakes and the three sites of the borehole; Table S2: The major nutrients, δD and δ^18^O values of the lake and glacier borehole meltwater; Table S3: The concentration of dissolved trace elements of the lake and glacier borehole meltwater; Figure S1: Welch’s *t*—test showing significant differences in the bacterial communities at the genus level between the samples from the lakes and glacier borehole meltwater. Statistical significance is indicated by asterisks: * 0.01 < *p* value ≤ 0.05; ** 0.001 < *p* value ≤ 0.01; *** *p* value ≤ 0.001; Figure S2: The values of δD and δ^18^O, and the relative abundance of characterized bacterial groups and the bacterial groups >5% with the depth of the glacier borehole meltwater.
