# Supplementary material for: Bacterial Distribution in the Glacier Borehole Meltwater on the Eastern Broknes Peninsula of the Larsemann Hills and Adjacent Lake Water, East Antarctica
Source: Microorganisms. 2025 Mar 18;13(3):679. doi: 10.3390/microorganisms13030679 (PMC11945024; doi:10.3390/microorganisms13030679)
Supplement: Supplementary file 1 [file microorganisms-13-00679-s001.zip › Supplementary material S1.pdf]

Supplementary material

Table S1. Locations of sampling sites of the lakes and the three sites of the borehole.

Sites	Latitude	Longitude
Mochou Lake	69° 22′ 22.67″ S	76° 22′ 11.82″ E
Longquan Lake	69° 23′ 22.85″ S	76° 22′ 23.14″ E
Progress Lake	69° 24′ 02.62″ S	76° 23′ 35.49″ E
Qingcheng Lake	69° 24′ 10.49″ S	76° 21′ 24.24″ E
135.3 mbsf (Ice)	69° 28′ 12.31″ S	76° 20′ 42.83″ E
183 mbsf (Ice)	69° 28′ 12.30″ S	76° 20′ 42.90″ E
200 mbsf (Ice)	69° 28′ 12.27″ S	76° 20′ 43.03″ E

Note: mbsf: abbreviation for meters below the surface

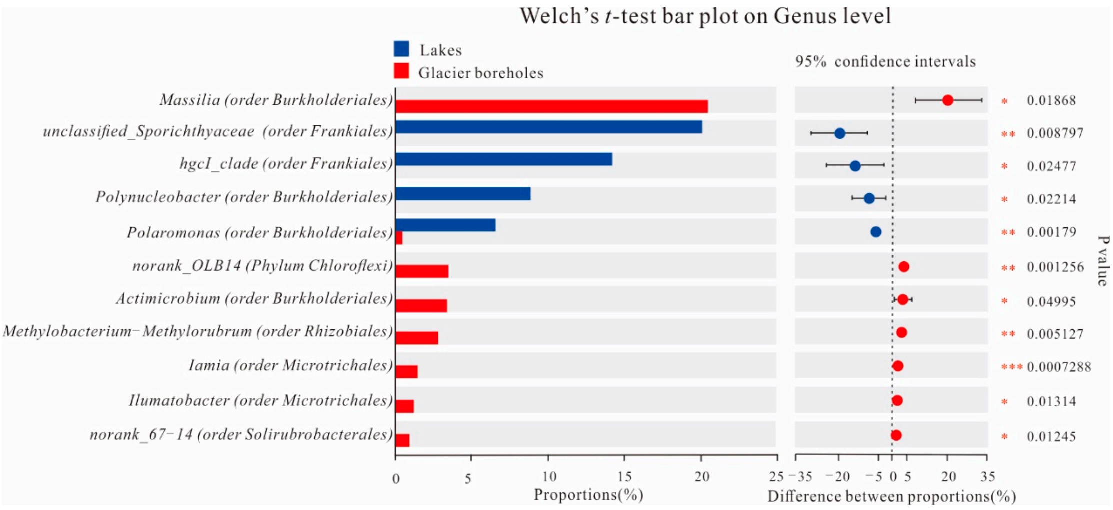

Figure S1. Welch's *t*-test showing significant differences in the bacterial communities at the genus level between the samples from the lakes and glacier borehole meltwater. Statistical significance is indicated by asterisks: * 0.01 < P value ≤ 0.05; ** 0.001 < P value ≤ 0.01; ***P value ≤ 0.001.

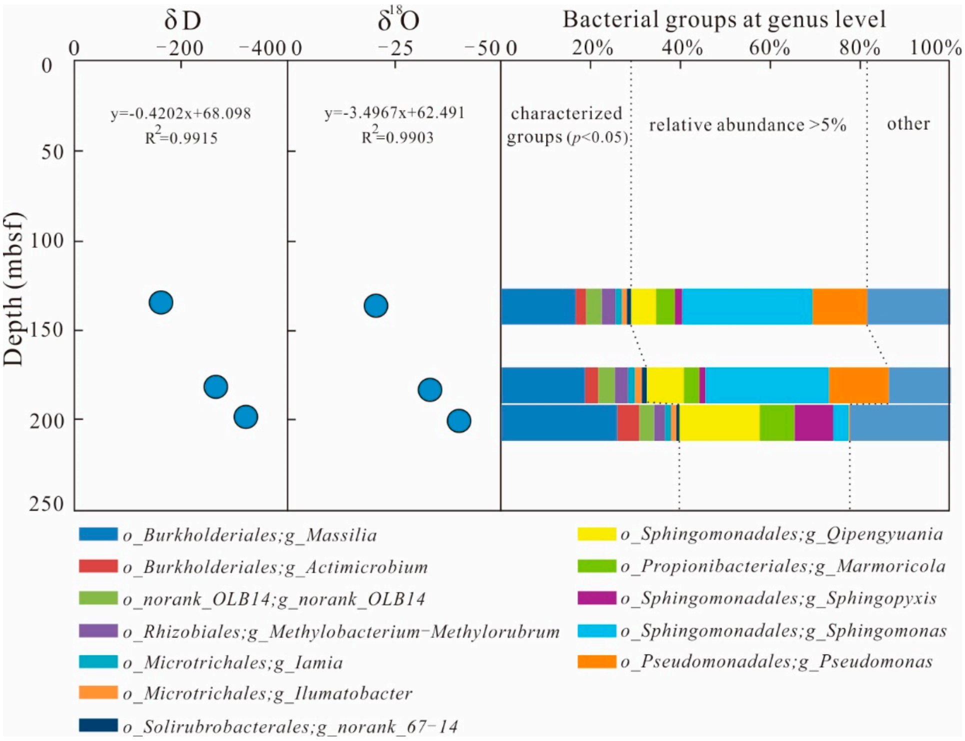

Figure S2. The values of δD and $\delta^{18}O$, and the relative abundance of characterized bacterial groups and the bacterial groups >5% with the depth of the glacier borehole meltwater.
